# Neuroimaging Findings in Nondemented Frail Individuals: A Systematic Review

**DOI:** 10.1002/jcsm.13719

**Published:** 2025-02-11

**Authors:** Hamid Harandi, Soheil Mohammadi, Ali Jahanshahi, Mahsa Dolatshahi, Sogol Alikarami, Rasa Zafari, Cyrus A. Raji

**Affiliations:** ^1^ School of Medicine Tehran University of Medical Sciences Tehran Iran; ^2^ Research Center for Antibiotic Stewardship and Antimicrobial Resistance, Imam Khomeini Hospital Complex Tehran University of Medical Sciences Tehran Iran; ^3^ School of Medicine Guilan University of Medical Sciences Rasht Iran; ^4^ Mallinckrodt Institute of Radiology Washington University in St. Louis Saint Louis Missouri USA

**Keywords:** frailty, magnetic resonance imaging, neuroimaging, systematic review, white matter hyperintensities

## Abstract

**Background:**

Frailty is a chronic condition characterised by the progressive decline of multiple physiological functions. There is a critical need to investigate neuroimaging findings in nondemented frail individuals to better understand the underlying mechanisms and implications of frailty on brain health. This paper is aimed at reviewing neuroimaging studies assessing brain changes in nondemented frail individuals to understand the neuropsychological basis of frailty.

**Methods:**

A systematic review was conducted on studies focusing on neuroimaging modalities in frailty, including MRI, fMRI, DTI and PET. The review was based on PRISMA instructions and a two‐step screening process. The studies evaluating neuroimaging findings of nondemented frail individuals, regardless of publication time or participant age, were included. Data were extracted from the included studies, and the quality of the studies as well as risk of bias was assessed.

**Results:**

Out of 1604 studies screened, 22 eligible studies were included. Out of these, 10 studies had good quality, while others had fair quality according to the Newcastle Ottawa scale (NOS). Of these studies, 18 used Fried criteria or a modified version of it to diagnose frailty, while the Edmonton frailty score (EFS), Rockwood and Mitnitski frailty index and frailty index (FI) were implemented by the remaining studies. The MRI findings indicated significant differences in brain structure between nondemented frail and robust individuals, including an increased number and size of white matter hyperintensities, reduced grey matter volume, higher cerebrospinal fluid (CSF) volume and increased number of cerebral microbleeds (CMBs) in frail participants compared to the robust ones. The studies showed no significant difference between at‐risk and robust groups regarding total intracranial volume (TIV). The number of CMBs was associated with prefrailty status and its severity. fMRI studies showed decreased intranetwork mean functional connectivity (FC) in nondemented frail individuals. DTI studies showed lower fractional anisotropy (FA), higher axial diffusivity (ad) and higher radial diffusivity (RD) in the nondemented frail group. The PET scan study showed that mean cortical beta‐amyloid level was not associated with FI, but the accumulation of beta‐amyloid in the anterior and posterior putamen and precuneus region significantly correlated with frailty and its severity.

**Conclusion:**

The study reveals significant differences in brain structures between nondemented frail and robust individuals, including increased white matter hyperintensities and reduced grey matter volume. These differences suggest that vascular changes and brain atrophy in nondemented frail individuals may contribute to cognitive impairment and dementia in the future.

## 1 Introduction

Frailty is a chronic state of progressive deterioration in multiple physiological functions, leading to adverse health outcomes, disabilities and mortality, particularly in geriatric health [1]. Physical frailty (PF) is the condition of having specific physical characteristics, which is further discussed [2, 3]. There are multiple tools available for assessing frailty status [4]. Fried criteria mainly focus on physical aspects and identify frailty by the presence of three or more of five frailty phenotypes: unintentional weight loss (10 lbs in the past year), exhaustion, diminished strength (assessed through grip strength), slowness in gait and decreased levels of physical activity [1, 5]. Prefrailty, on the other hand, is a clinically latent process in individuals with a Fried criteria score of 1–2, predisposing them to frailty [6, 7]. Patients not falling into either of these two categories are classified as the robust group, predominantly characterised by a 0 score on the Fried criteria. The frailty index (FI) considers frailty as an accumulation of deficits in multiple older adults' health‐related domains, such as signs and symptoms of a disease, cognitive disorders, functional disabilities, psychological problems and laboratory abnormalities [1, 8, 9].

Frailty has been a burden due to its consequences, such as hospitalisation, higher demand for advanced care and falls [10]. Despite its significance in geriatric health and the complicated aetiology of this condition, no specific standard definition has been established for frailty. Studies have suggested various scaling instruments to clinically evaluate individuals regarding the stages of frailty [11]. Yet, it is crucial that different methods be utilised to unravel the underlying mechanism of this condition. Previous publications have illustrated an association between PF and cognitive impairments [2, 12]. These findings insinuate that cognitive decline due to various neurodegenerative alterations might have a predictive value for PF. Hence, investigating frail individuals for neurodegenerative changes appears to be a crucial step forward.

Magnetic resonance imaging (MRI) provides a substantial amount of data on macroscopic brain structural alterations, namely white matter (WM) and grey matter (GM) volumes, as well as cerebrovascular changes such as microbleeds and small vessel disease [13, 14]. These findings enable us to investigate the potential associations between frailty and neurodegenerative processes. On the other hand, functional neuroimaging techniques can give insights into activity and connectivity in different brain areas. Functional MRI (fMRI) uses signals from blood flow to evaluate the brain regions' functional connectivity (FC) or activation patterns during a task or in a resting state [15]. Furthermore, in positron emission tomography (PET), the activity of the various brain regions is mapped according to the measured cerebral uptake of radioactive substances [16]. Since the accumulation of beta‐amyloid has an essential role in the pathophysiology of cognitive impairment in Alzheimer disease, PET scans can tremendously aid researchers in tracing Alzheimer's disease pathology [17]. Additionally, advanced methods such as diffusion tensor imaging (DTI) have been introduced to assess microstructural alterations in the CNS based on the diffusion of the water molecules. Diffusion parameters, such as fractional anisotropy (FA), mean diffusivity (MD), axial diffusivity (ad) and radial diffusivity (RD), represent a measure of microstructural integrity, membrane density and parallel or perpendicular diffusion of the water molecules toward the neuron fibre, respectively [18]. Changes in each parameter can suggest a specific alteration in neuronal integrity, that is, decreased FA and higher values of MD might be due to a demyelinating process [19].

As mentioned above, evidence shows significant associations between frailty and cognitive impairments. The review by Sousa‐Fraguas et al. [20] focused on 12 studies assessing frailty and cognitive function in patients with Parkinson's disease (PD), most of which showed cognitive impairments in PD patients with frailty. Besides, another study conducted by Karanth et al. [21] showed that in the frail and prefrail individuals the global cognitive scores were lower than in the robust ones, while the prevalence of self‐reported memory complaint was higher in the frail and prefrail groups compared with controls.

The mentioned findings demonstrate the importance of investigating the association between neurodegenerative alterations and frailty. Accordingly, in this paper, we aim to systematically review the neuroimaging studies that assess CNS changes in nondemented frail individuals. By gathering the results from different neuroimaging modalities and understanding the relation between frailty and nondementia cognitive impairments, we can depict the possible implications of frailty in the brain and obtain comprehensive insights about the neuropsychological basis of this condition.

## 2 Method

We performed a comprehensive systematic review based on Preferred Reporting Items for Systematic Reviews and Meta‐Analyses (PRISMA) instructions [22]. The protocol was registered in the International Prospective Register of Systematic Reviews (PROSPERO) (https://www.crd.york.ac.uk/prospero/display_record.php?ID=CRD42023398553). We aimed to systematically review the studies focusing on different neuroimaging modalities, such as MRI, fMRI, DTI and PET in frailty up to 30 January 2024, and conducted a two‐step screening process to include eligible studies.

### 2.1 Search Strategy and Study Selection

We systematically searched PubMed and Embase databases initially on 7 February 2023, and then, we updated our systematic search on 30 January 2024 with the mentioned keywords related to the topic (Table S1). We also manually searched other resources, such as websites and citations, to find any study that might have been missed. After removing duplicate articles, the first step of the screening based on the title and abstract, a full‐text screening was carried out by two reviewers independently, and any conflicts or discrepancies were resolved through discussion.

### 2.2 Inclusion and Exclusion Criteria

All studies evaluating the neuroimaging findings of frail individuals through MRI, fMRI, DTI and PET until 30 January 2024 were included regardless of their publication time and age of participants. Studies evaluating the neuroimaging findings of frailty in dementia patients, non‐English studies, animal studies, conference abstracts, letters to editors, reviews and case studies were excluded.

### 2.3 Data Items

The following items were extracted from included studies by two reviewers independently, and discrepancies were resolved through discussion by a third reviewer: first author's name, year of publication, study design, country, study groups, participants' age and sex, modality of imaging, frailty measurement criteria, the tools by which the dementia was excluded, parameters that the study groups were matched for, imaging acquisition protocol, covariates in different modalities and neuroimaging findings.

### 2.4 Quality and Risk of Bias Assessment

The quality of the included studies was assessed by two reviewers separately using the Newcastle Ottawa scale (NOS) for nonrandomised studies consisting of sample selection, comparability of the groups and ascertainment of the exposure [23]. The NOS rating ranges from 0 to 9, including 4 points for selection, 2 points for comparability and 3 points for outcomes. Accordingly, the studies were categorised into good, fair and poor. Studies with a score of 7–9 have the lowest risk of bias and the highest quality, while studies with a score of less than 4 have the highest risk of bias and the lowest quality. Studies with a score of 4–6 have a moderate risk of bias and quality.

Also, the risk of bias for each study was assessed according to a study from Viswanathan et al. [24] for selection, performance, attrition, detection and reporting biases. Any conflicts were solved through discussion with a third reviewer.

## 3 Results

### 3.1 Study Selection

**FIGURE 1:**
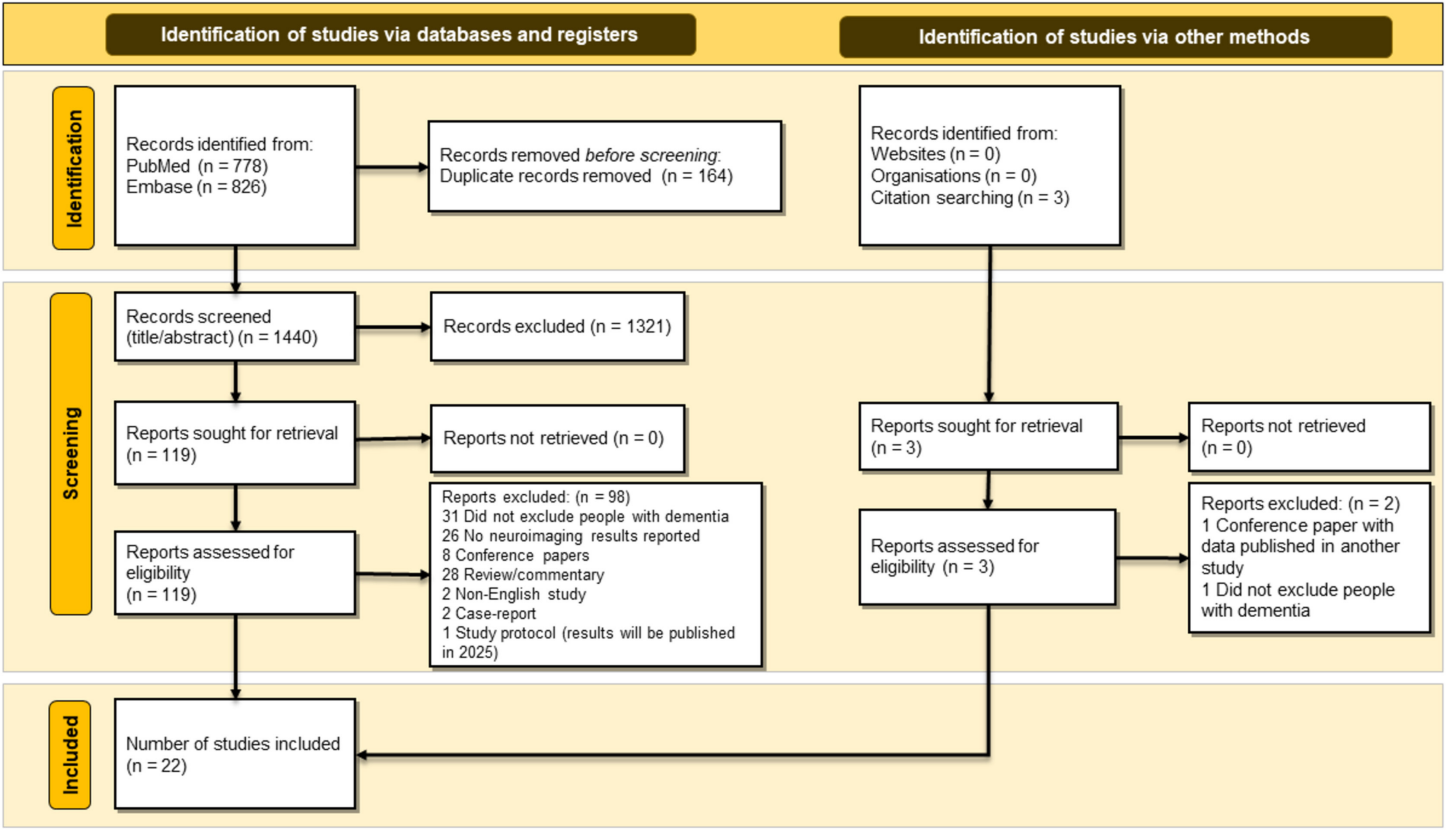
Flowchart of study selection.

We extracted 1604 studies from databases. After removing duplicates, 1440 studies underwent the title/abstract screening process, which yielded 120 studies for full‐text review. Ninety‐seven studies were excluded because they did not meet our eligibility criteria: not excluding participants with dementia (*n* = 31), not reporting neuroimaging results (*n* = 26), conference papers (*n* = 8), reviews or commentaries (*n* = 28), studies in languages other than English (*n* = 2), case studies (*n* = 2) and a protocol. In addition, we identified three studies by citation searching, of which one conference paper was excluded due to the data published in another original study and another did not exclude people with dementia. Finally, 22 studies [3, 25, 26, 27, 28, 29, 30, 31, 32, 33, 34, 35, 36, 37, 38, 39, 40, 41, 42, 43, 44, 45] that met the inclusion criteria of our study were included. Figure 1 details the process of the study selection.

### 3.2 Characteristics of the Included Studies

Of the included studies, 16 were cohort [25, 26, 27, 28, 29, 31, 32, 33, 36, 38, 39, 40, 42, 43, 44, 45], and six [3, 30, 34, 35, 37, 41] were case–control studies. Regarding the modality of neuroimaging, 16 studies [3, 25, 26, 27, 28, 29, 31, 34, 35, 38, 39, 40, 42, 43, 44, 45] reported MRI results, five [25, 28, 37, 41, 44] used DTI, three studies [30, 32, 33] implemented fMRI and one [36] used PET. The sample size of the studies varied vastly from 40 [34] to 1754 [28], and their ages ranged from 68.3 ± 6.2 [43] to 81.3 ± 8.0 [41] in the frail groups and from 60.34 ± 7.28 [27] to 78.50 [30, 40] in the robust groups. Furthermore, four studies [30, 40, 41, 42] matched the study groups for age, two of which [30, 40] also matched them for gender and another study [42] matched the study groups for their sex.

Seventeen studies [3, 25, 26, 27, 28, 29, 30, 31, 32, 33, 35, 37, 40, 41, 42, 43] implemented Fried criteria to assess the stage of frailty, of which four [31, 32, 33, 42] used the modified version. Four studies [36, 38, 39, 44] used FI, and one study [34] assessed frailty using the Edmonton frailty scale (EFS). Almost half of the included studies excluded cognitive impairments in the participants based on Mini‐Mental State Examination (MMSE) scores [25, 26, 27, 29, 30, 31, 32, 33, 34, 40], whereas others used different methods for excluding dementia [28, 36, 41]. However, only two studies [28, 41] excluded mild cognitive impairment (MCI). Eleven studies [3, 25, 26, 29, 30, 31, 32, 33, 34, 37, 40] reported MMSE scores of their study population, which ranged from 20.8 [34] to 29 [29, 31, 32, 33]. More information on the studies' characteristics can be obtained from Table 1.

**TABLE 1:** Characteristics of included studies.

Study	Study design	Country	Study groups (frailty score)	Sample size F/M	Age (years) mean ± SD	Mean MMSE ± SD	Modality	Frailty measurements	Cognitive status	Matched for
Avila‐Funes, 2016	Cohort	Mexico	Robust	51/92	75.0 ± 5.0	27.2 ± 2.1	MRI, DTI	Fried criteria	MMSE > 15	—
			Frail	19/14	76.0 ± 5.7	26.4 ± 2.7				
Chen, 2015	Cohort	Taiwan	Robust (0)	119/140	62.5 ± 7.7	26.9 ± 2.9	MRI	Fried criteria	Dementia excluded based on MMSE score	—
			Prefrail (1–2)	89/89	65.2 ± 9.0	25.8 ± 3.4				
			Frail (≥ 3)	9/10	73.6 ± 7.4	23.1 ± 3.8				
Chung, 2016	Cohort	Taiwan	Robust (0)	348/263	60.34 ± 7.28	—	MRI	Fried criteria, CHS score	Score > 24 in well‐educated subjects MMSE score > 14 in less‐educated subjects	—
			Prefrail (1–2)	173/146	65.28 ± 9.00					
			Frail (≥ 3)	16/16	74.90 ± 7.86					
Ducca, 2022	Cohort	United States	Robust (< 3)	954/671	76.0 ± 4.7	—	MRI, DTI	Fried criteria	Participants who did not meet criteria for MCI or dementia according to expert adjudications	—
			Frail (≥ 3)	87/42	78.5 ± 5.5					
Kant, 2019	Cohort	Netherlands	Robust (0)	12/43	70 ± 4	29	MRI	Fried criteria	MMSE > 24	—
			Prefrail (1–2)	24/61	72 ± 5	29				
			Frail (≥ 3)	16/14	74 ± 5	28				
Suárez‐Méndez, 2020	Case–control	Spain	Robust	21/13	78.50	27.0	MEG, MRI	Fried criteria	MMSE > 24	Age and gender
			Frail	15/5	81	26.0				
Kant, 2018	Cohort	Netherlands	Robust (0)	24/51	71.6 ± 4.5	29	MRI	Modified version of Fried criteria	MMSE > 24	—
			Prefrail (1–2)	37/70	72.3 ± 5.0	29				
			Frail (≥ 3)	19/13	74.7 ± 5.4	28				
Lammers, 2020	Cohort	Germany	Robust (0)	19/41	70.5	29	fMRI	Modified version of Fried criteria	MMSE > 24	—
			Prefrail (1–2)	37/33	71.5	29				
			Frail (≥ 3)	8/5	74.0	28				
Lammers, 2022	Cohort	Germany	Improving	10/9	73	28	fMRI	Modified version of Fried criteria	MMSE ≥ 23	—
			Stable	25/52	71	29				
			Progressing	10/14	71	29				
Li, 2021	Case–control	China	Nonfrail (≥ 8)	7/15	75.0 ± 6.2	24.6 ± 5.3	MRI	EFS	MMSE > 15	—
			Frail (< 8)	12/6	77.9 ± 4.3	20.8 ± 4.7				
Maltais, 2019	Case–control	France	Robust (0)	71/42	74.9 ± 4.1	—	MRI	Fried criteria	Dementia excluded	—
			Prefrail (1–2)							
			Frail (≥ 3)							
Maltais, 2019	Cohort	France	—	162/269	74.7 ± 4.3	—	PET	Frailty index	CDR < 1	—
Maltais, 2020	Case–control	France	Robust (0)	136/91	74.7 ± 3.9	28.1 ± 1.5	DTI	Fried criteria	Dementia excluded	—
			Prefrail (1–2)							
			Frail (≥ 3)							
Siejka, 2020	Cohort	Australia	Lower frailty	85/108	69.5 ± 6	—	MRI	Rockwood and Mitnitski frailty index	Dementia excluded	—
			Higher frailty	87/108	74.5 ± 7.1					
Siejka, 2017	Cohort	Australia	—	172/216	72.0 ± 7.0	—	MRI	Cumulative deficit model of frailty index	Dementia excluded	—
Sourdet, 2021	Cohort	France	—	—	—	—	Amyloid PET, MRI	Fried criteria	MMSE > 20	—
Suárez‐Méndez, 2021	Cohort	Spain	Robust	21/13	78.50	27.00	MEG, MRI	Fried criteria	MMSE ≥ 24	Age and gender
			Frail	15/5	81.00	26.00				
Sugimoto, 2019	Case–control	Japan	Healthy older adults	120/77	75.5 ± 5.6	28.2 ± 2.1	MRI	Fried criteria	Alzheimer disease ruled out based on criteria of the National Institute on Aging‐Alzheimer's Association (NIA/AA)	—
			With CF	86/50	73.4 ± 5.2	25.3 ± 2.5				
Tian, 2020	Case–control	United States	Robust (0)	185/177	70.2 ± 6.9	—	DTI	Fried criteria	MCI excluded based on Peterson criteria Dementia excluded based on DSM‐III‐R Alzheimer disease excluded based on NINCDS‐ADRDA criteria	Age
			Prefrail (1–2)	168/111	77.2 ± 7.8					
			Frail (≥ 3)	21/8	81.3 ± 8.0					
Nishita, 2019	Cohort	Japan	Robust (0)	137/192	72.7 ± 5.1	—	MRI	Modified version of Fried criteria	Dementia excluded	Age and sex
			Prefrail (1–2)	224/204	74.6 ± 5.8					
			Frail (≥ 3)	54/24	79.2 ± 6.3					
Zhao, 2021	Cohort	China	Robust (0)	206/161	64.3 ± 5.9	—	MRI	Fried criteria	Alzheimer disease excluded	—
			Prefrail (1–2)	199/111	66.5 ± 6.9					
			Frail (≥ 3)	29/20	68.3 ± 6.2					
Isernia, 2023	Cohort	Italy	Robust (0)	17/17	71.5, 6.75^a^	27.0, 3.5^a^	MRI	Fried criteria	Severe dementia excluded	—
			Prefrail (1–2)	27/18	75.0, 10.0^a^	26.0, 3.0^a^				
			Frail (≥ 3)	14/3	78.0, 11.0^a^	25.7, 3.9^a^				
Gutiérrez‐Zúñiga, 2023	Cohort	Ireland	Robust (FI < 0.10)	143/152	66.9 ± 7.0	—	MRI, DTI	A self‐reported 32‐item FI	Neurodegenerative diseases excluded	—
			Prefrail (FI 0.10–0.24)	103/87	70.7 ± 7.0					
			Frail (FI ≥ 0.25)	23/15	74.3 ± 6.9					

Abbreviations: CDR, Clinical Dementia Rating scale; CHS, Cardiovascular Health Study frailty score; CN, cognitively normal; DSM‐III‐R, Diagnostic and Statistical Manual, third edition revised; DTI, diffusion tensor imaging; EFS, Edmonton frailty scale; fMRI, functional magnetic resonance imaging; IST, Isaacs Set Test; MCI, mildly cognitively impaired; MEG, magnetoencephalography; MMSE, Mini‐Mental State Examination; MRI, magnetic resonance imaging; NINCDS‐ADRDA, National Institute of Neurological and Communicative Disorders and Stroke–Alzheimer's Disease and Related Disorders Association; PET, positron emission tomography.

^a^ Median, IQR.

### 3.3 Quality and Risk of Bias Assessment

Among the cohort studies, 10 [26, 27, 28, 31, 38, 39, 42, 43, 44, 45] had a good quality according to the NOS (scores 7–9). All case–control studies and other cohorts had either fair quality (*n* = 6). Regarding the risk of bias, almost all of the studies were considered low risk for selection and performance bias. Except for five [37, 38, 39, 44, 45], all included cohort studies were at high risk for attrition bias. Similarly, all were at high risk for reporting bias, except four studies [37, 41, 44, 45]. Detailed information on quality and risk of bias assessment is illustrated in Tables S2–S4.

### 3.4 Neuroimaging Findings

**FIGURE 2:**
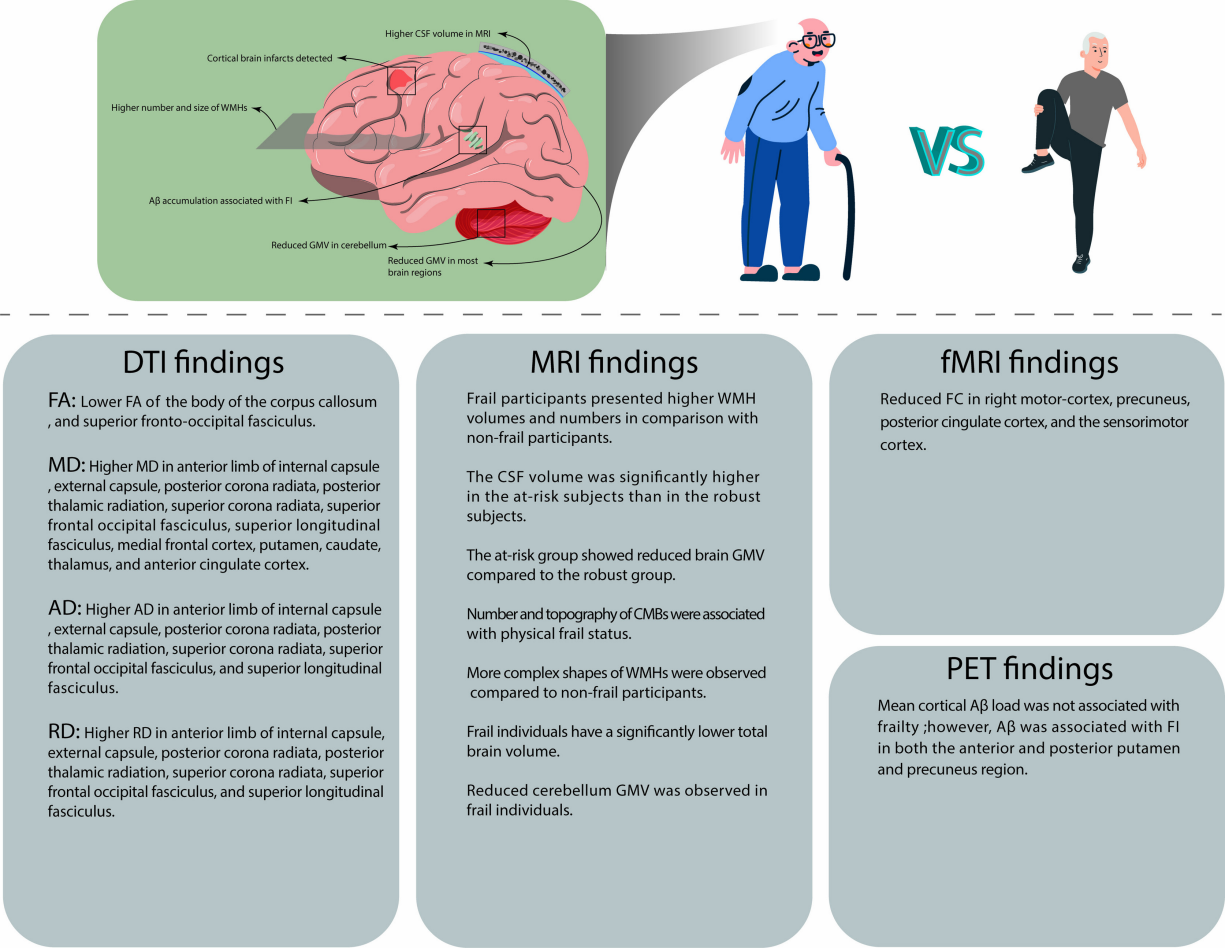
An overview of neuroimaging findings in frail patients compared with normal individuals. GMV, grey matter volume; DTI, diffusion tensor imaging; MRI, magnetic resonance imaging; fMRI, functional magnetic resonance imaging; PET, positron emission tomography; FA, fractional anisotropy; MD, mean diffusivity; ad, axial diffusivity; RD, radial diffusivity; CSF, cerebrospinal fluid; Aβ, amyloid beta; CMB, cerebral microbleed; WMH, white matter hyperintensity; FC, functional connectivity; FI, frailty index.

The findings with different imaging modalities (structural MRI, fMRI, diffusion MRI and PET scan) are summarised in later section. Figure 2 provides a brief summary of the findings from the included studies.

#### 3.4.1 Structural MRI Studies

As illustrated in Table 2, in one of the included MRI studies conducted by Chen et al. [26], no significant difference was detected between at‐risk and robust groups regarding total intracranial volume (TIV). However, Sugimoto et al. [3] reported lower intracranial volume (ICV) in the cognitive frailty group, and Kant et al. [31] found a decreased total brain volume (TBV) in frail individuals.

**TABLE 2:** A summary of findings of studies on MRI.

Study	MR field strength and sequence	Measures	Brain areas	Covariates	White matter alterations	Grey matter alterations	CSF alteration	Whole brain alterations	Other correlations
Avila‐Funes, 2016	1.5 T FLAIR, T1	WHV	—	Age, sex, educational level, myocardial infarction, angina pectoris, stroke or hypercholesterolaemia, hypertension, diabetes, smoking status, cognitive status	Higher WHV in frail older adults compared with nonfrails	—	—	—	—
Chen, 2015	3 T T1, T2‐FLAIR	GMV, WMV, CSF volume, WMH, TIV	—	Age, sex, Charlson comorbidity index, cognitive function, mood and depressive symptoms, educational years, BMI, blood cell count and biochemistry (fasting blood sugar, total hypercholesterolaemia, albumin, total protein, uric acid, serum creatinine, Hs‐CRP, urine analysis and dual‐energy X‐ray absorptiometry)	No difference in WMH quantification and WM volume	No difference in GMV At‐risk group showed reduced GMV, compared to the robust group, in 13 brain regions including the cerebellum, hippocampi, middle frontal gyri, right inferior parietal lobule, precentral gyrus, left insula, anterior cingulate and middle occipital gyrus	Higher CSF volume in the at‐risk (prefrail and frail) subjects compared with the robust group	No difference in TIV volume	Slowness, weakness and low activity involved reduced GMV in the cerebellum. Weight loss was associated with both GMV reduction (the right postcentral gyrus) and increase (the right posterior cingulate) topographically different from frailty‐related regions
Chunga, 2016	3 T T2‐FLAIR	CMB, number of lacune, WMH	‐ Deep (basal ganglia, thalamus, internal capsule, external capsule, corpus callosum and deep/periventricular white matter), lobar (fontal, parietal, temporal and occipital regions) ‐ Infratentorial categories (brainstem and cerebellum)	Age, sex, educational levels, smoking status, coronary artery disease, CKD, hypertension, DM, hyperlipidaemia, height, weight, BMI, cognitive function, mood and depressive symptoms	—	—	—	—	Both number and topography of CMB were associated with physical frail status, independent of age and vascular risk factors. The incidence and number of CMBs in the deep or infratentorial regions were associated with the severity of physical frailty. CMBs located in the lobar regions, either mixed with deep/infratentorial or strictly lobar CMB, were not associated with the severity of the physically frail status. The number of lacunes was significantly associated with the severity of the physical frail status. CMB located in the brainstem was significantly associated with physical frailty. The presence of CMBs in the brainstem was significantly associated with the severity of physical frailty; the presence of CMB in the brainstem was revealed as an independent factor associated with the presence of weakness
Ducca, 2022	3 T T2, FLAIR	WHV	—	Age, sex, race, educational levels, hypertension, coronary artery disease, diabetes, current smoking, stroke, cognitive status, BMI, APOEe4 status	Individuals with physical frailty have greater WHV and white matter microstructural abnormalities than nonfrails. This relationship was observed in participants without a history of stroke or dementia but did not persist when analyses were restricted to cognitively normal individuals, that is, the group of participants without MCI or dementia. WHV was significantly associated with new‐onset frailty over a 7‐year follow‐up period, even among cognitively normal adults. These results were consistent in Black and White participants and in men and women. Unlike WHV, general measures of white matter microstructural integrity were not associated with the risk of future frailty. However, secondary analyses did find that the microstructural integrity of specific white matter tracts was associated with future frailty	—	—	—	—
Kant, 2019	3 T T1, FLAIR, DWI	WHV, WMH shape, lacunar infarcts	—	Age, sex, hypertension, cognitive status, depressive symptoms, diabetes, BMI, obesity, smoking status, stroke, American Society of Anaesthesiologists score	Frail and prefrail participants had a higher WHV compared to nonfrail participants. Prefrail participants showed a more complex shape of periventricular and confluent WMH compared to nonfrail participants	—	—	—	No between‐group differences were found in shape features of deep WMH, cerebral perfusion or presence of lacunar infarct
Kant, 2018	3 T T1, FLAIR	TBV, GMV, WHV, WM volume, presence of a cortical brain infarct	—	Age, gender, BMI, DM, smoking status, hypertension, hypercholesterolaemia, cognitive status, American Society of Anaesthesiologists score	Higher WHV in frail individuals	Lower GMV in frail individuals	—	Lower TBV in frail individuals; frail individuals showed a lower global cortical thickness compared to prefrail and nonfrail individuals; however, no regional clusters of a lower cortical thickness were found	More cortical infarcts in frail individuals; individual frailty components showed a relation with a lower global grey matter volume, but this did not reach statistical significance. Furthermore, prefrail individuals had more cortical brain infarcts compared to nonfrail individuals
Li, 2021	3 T T1, T2, DWI	GMV, WMV, CSFV, MYV, BPV, ICV, GMF, WMF, MYF, GMV/ICV, WMV/ICV, CSF/ICV, MYV/ICV	—	Age, sex, BMI, educational level, cognitive status	—	Multiple regional GM volumetry showed significant correlations with the EFS. No significant correlation was found between the EFS and regional GM relaxometry. Both global volumetry and global relaxometry showed strong correlations with some domain scores of the EFS. Some regional GM volumetry showed strong negative correlations with the functional performance score. Significant negative correlations were identified between the functional performance score and the GMV of Rolandic, olfactory, rectus, cingulate, hippocampus, parahippocampal and precuneus. No notable correlations were found between regional GMV and the cognition score, general health status score, functional independence score, medication use score, nutrition score, mood score or continence score. Significant negative correlations were found between the functional independence score and the T1 value of caudate. No notable correlations were identified between the other scores and regional GM T1 values. Significant negative correlations were identified between the functional independence score and the T2 values of insula and caudate. No notable correlations were found between the other scores and regional GM T2 values. No significant correlations between the domain scores of the EFS and regional GM PD values were identified	—	—	—
Maltais, 2019	—	WHV, WMH progression	—	Age, sex, educational level, DM, hypertension, hypercholesterolaemia	A significant increase in likelihood of increasing 1 point on the frailty phenotype score over 3 years with higher baseline WMH. For the progression of WMH, no significant associations were found for the continuous variable as well as the dichotomous variable (fast vs. slow progression of WMH) with frailty evolution	—	—	—	—
Siejka, 2020	1.5 T T1, T2, FLAIR, GRE	WMH, SI, microbleeds	—	Age, sex, educational level	Higher baseline WMH was associated with a greater progression in frailty. WMH was significantly associated with progressively worsening frailty. A higher burden of baseline WMH was more strongly associated with progression of frailty	—	—	—	SI and microbleeds were not significantly associated with progression of frailty
Siejka, 2017	1.5 T T1, T2, FLAIR, GRE	WMH, SI, CMB	—	Age, sex, educational level	WMH in unadjusted models was positively associated with higher frailty scores. In final models including all brain variables, higher burden of WMH was independently associated with a higher frailty score. In fully adjusted models, WMH was associated with the motor index	—	—	—	SI and CMB in unadjusted models were positively associated with higher frailty scores. In final models including all brain variables, higher burden of SI or CMB was not associated with a higher frailty score
Suárez‐Méndez, 2021	0.23 T T1	MEG	Whole brain	Age, gender, hypertension, DM, dyslipidaemia, COPD/asthma, presence of osteoporosis, vertebral fracture, low Vitamin D, AF, IHD, cancer, incontinence, functional status, neuropsychological status	—	—	—	Significant group differences in relative power were found in the classical alpha (mu) and SMR frequency bands. We found no significant group differences in theta nor beta frequencies after multiple comparison correction found in the RH affecting posterior parietal areas (e.g., AG and SPL), the primary somatosensory cortex and the superior occipital cortex. Significant bilateral differences involved areas of the primary motor cortex. These regions comprise those classically associated with traditional generators of mu activity; thus henceforth, we will refer to our results within this range as mu oscillations Significant group differences were found in SMR relative power after multiple comparison correction. Frail participants exhibited increased SMR relative power in comparison to their robust peers. The strongest differences were found in frontal areas of the RH, including the premotor cortex, the supplementary motor area and the paracingulate gyrus	—
Sugimoto, 2019	1.5 T T1, T2, FLAIR	ICV, WMH, parenchyma volume, WMH/PAR	—	Age, sex, BMI, educational level, cognitive function, functional status, smoking status, drinking status, hypertension, DM, hyperlipidaemia, coronary artery disease, CRP, BNP, homocysteine, eGFR	Higher WMH and WMH/PAR volumes in CF individuals. WMH/PAR volume was shown to be highest among those with minimal cognitive impairment and PF. The PF/CN, pre‐PF/MCI and PF/MCI groups had significantly higher WMH/PAR volumes compared to the non‐PF/CN group. PF subjects had higher WMH volumes compared to non‐PF subjects among patients without MCI	—	—	Lower IC and PAR volumes in CF individuals	—
Nishita, 2019	3 T T1	GMV	—	Age, sex, education level, marital status, conditions of habitation (living alone, living with others), employment (employed, unemployed), current smoking status, hypertension, stroke, heart disease, DM, dyslipidaemia, presence of osteoporosis, chronic bronchitis and knee arthropathy	—	Volumes of GM of each region did not differ significantly among physically frail, prefrail and robust groups	—	—	Weakness and slowness were significantly and negatively associated with the grey matter volumes of specific regions. Weakness was significantly associated with reduced GMV in the right anterior hippocampal and amygdala clusters and the bilateral fusiform gyrus. Regions significantly associated with slowness overlapped with regions significantly associated with weakness: the bilateral anterior hippocampus and amygdala and fusiform gyrus. Slowness was associated with a reduced volume of more extensive brain regions, including the bilateral medial prefrontal and orbitofrontal cortex, inferior frontal gyrus, primary somatosensory cortex, insula, superior temporal sulcus and cerebellum
Zhao, 2021	3 T T1, T2, FLAIR, DWI, SWI	WMH, lacunes, CMB, EPVS‐BG	—	Age, sex, weight, education level, smoking, and alcohol drinking status, hypertension, stroke, coronary heart disease, DM, hyperlipidaemia, kidney disease, arthritis, migraine, depressive symptoms, sleep quality	The severity of the WMH was correlated with frailty and sleep quality	—	—	—	Participants with a higher CSVD burden were more likely to be frail. The severity of the EPVS‐BG and the number of lacunes were correlated with frailty and sleep quality. Sleep quality played a partial mediating role in the association between CSVD burden and physical frailty
Isernia, 2023	3 T T1‐3D, FLAIR, T2	Brain morphometry, WMH	Whole brain	Sex, age, education level, memory, attention, language	No notable associations between the frailty score and the overall volume of WMH	Frailty was shown to be correlated with decreased cortical thickness in the left supramarginal gyrus and the right rostral middle frontal gyrus	—	—	No notable associations between the frailty score and the volumes of subcortical areas
Gutiérrez‐Zúñiga, 2023	3 T T1‐3D, DTI	Grey matter measures (cortical and subcortical) and white matter tracts	Cortex, basal ganglia and each of the Desikan–Killiany regional volumes	Age, sex, medical history (number of regular medications, vascular diseases, cancer and smoking history), physical exercise in metabolic equivalents and quality of life measured by the CASP‐12 scale	—	—	—	The FI exhibited a negative correlation with the total volume of the cortex	The basal ganglia measurements showed a negative correlation between thalamic volumes and the FI

Abbreviations: AF, atrial fibrillation; AG, angular gyrus; BG, basal ganglia; BMI, body mass index; BNP, B‐type natriuretic peptide; BPV, brain parenchymal volume; CF, cognitive frailty; CKD, chronic kidney disease; CMB, cerebral microbleeds; CN, cognitively normal; COPD, chronic obstructive pulmonary disease; CRP, C‐reactive protein; CSF, cerebrospinal fluid; CSFV, cerebral spinal fluid volume; CSVD, cerebral small vessel disease; DAN, dorsal attention network; DM, diabetes mellitus; DWI, diffusion weighted imaging; EFS, Edmonton frailty scale; eGFR, estimated glomerular filtration rate; EPVS, enlarged perivascular spaces; FLAIR, fluid‐attenuated inversion recovery; FPN, frontoparietal network; GMF, grey matter fraction (GMV/BPV); GMV, grey matter volume; GRE, gradient echo sequences; hs‐CRP, high sensitivity C‐reactive protein; ICV, intracranial volume; IFG, inferior frontal gyrus; IHD, ischemic heart disease; MCI, mildly cognitively impaired; MEG, magnetoencephalography; MFG, middle frontal gyrus; MYF, myelin fraction (MYV/BPV); MYV, myelin volume; PAR, parenchyma; PD, proton density; pDMN, posterior default mode network; PF, physical frailty; RH, right hemisphere; SI, subcortical infarct; SMA, sensory motor area; SMG, supramarginal gyrus; SMR, sensorimotor rhythm; SPL, superior parietal lobule; SVD, small vessel disease; SWI, susceptibility weighted imaging; T, tesla; TBV, total brain volume; TIV, total intracranial volume; VAN, ventral attention network; WHV, white matter hyperintensity volume; WM, white matter; WMF, white matter fraction (WMV/BPV); WMH, white matter hyperintensity; WMV, white matter volume.

Most of the reported MRI results were focused on white matter hyperintensities (WMHs) and white matter volume (WMV) and grey matter volume (GMV). Twelve studies (two case–control and 10 cohort studies) [3, 25, 26, 27, 28, 29, 31, 35, 38, 39, 43, 45] reported data on WMHs, five of which [3, 25, 28, 29, 31] mentioned higher WMH volumes in the case groups. Sugimoto et al. [3] observed increased WMH volumes in PF and cognitive frailty. Two studies [35, 38] stated that higher baseline WMH volumes are associated with the progression of frailty, of which the study by Maltais et al. [35] observed complex‐shaped periventricular WMH in the prefrail group. The other studies [34, 40, 42] did not measure or report WMH. Ducca et al. [28] excluded MCI participants in their study and found nonsignificant differences in WMH in frail individuals with normal cognition. They also found that WMH volumes are correlated with new onset frailty over a 7‐year follow‐up [28].

One of the MRI studies reported lower GMV in frail individuals [31], while two studies [26, 42] mentioned no between‐group differences in GMV. Chen et al. [26] showed that in the at‐risk group, GMV was reduced in the cerebellum; hippocampi; middle frontal, precentral and middle occipital gyri; right inferior parietal lobule; left insula and anterior cingulate. Weight loss (a component of frailty) was shown to be linked to a decrease in GMV (specifically in the right postcentral gyrus) as well as an increase in GMV (specifically in the right posterior cingulate). These changes occurred in regions of the brain that are distinct from those associated with frailty [26]. Additionally, Li et al. [34] found that GMV in Rolandic, olfactory, rectus, cingulate, hippocampal and parahippocampal and precuneus regions was negatively correlated with functional performance scores. Likewise, Gutiérrez‐Zúñiga et al. [44] noted negative correlations between FI and total cortex and thalamic volumes. Furthermore, Nishita et al. noted that slowness and weakness significantly correlated with lower regional GMVs, detailed in Table 2.

Four studies [27, 38, 39, 43] demonstrated results on cerebral microbleeds (CMBs), and only one of them [27] included both lobar and deep CMBs and reported significant results, while the other three did not mention the type of CMBs. The study by Chung et al. [27] showed that the number of CMBs was associated with PF status. Moreover, unlike lobar microbleeds, CMBs in the brain stem were significantly correlated with PF and its severity and were independently associated with the presence of weakness [27]. Three studies [27, 29, 43] investigated the correlation between the number of lacunes and frailty, two of which [27, 43] mentioned a significant positive association.

#### 3.4.2 fMRI Studies

Three studies [30, 32, 33] evaluated the participants using fMRI, with FC as the main outcome. Mendez et al. [30] mentioned that intranetwork mean FC was decreased in frail individuals. They found reduced FC between the right angular gyrus and right motor, precuneus, posterior cingulate and sensorimotor cortices [30]. Additionally, FC was diminished between the right superior parietal lobule and premotor and anterior cingulate cortices and the frontal and left temporal poles, as well as between the bilateral supramarginal gyrus and left premotor, supplementary motor area (SMA), sensorimotor, right precuneus and bilateral cingulate cortices [30]. Lammers et al. [32] reported that FC in the SMA was the lowest in the frail group. Although FC in the pre‐SMA was not associated with frailty, it was significantly lower in the individuals with higher MMSE scores. In another study in 2022, Lammers et al. [33] focused on postoperative transitions in frailty stages and observed lower FC between SMA and left parietal cortex in subjects with undesirable outcomes, as well as increased pre‐SMA network connectivity in the patients with desirable postoperative transitions (improvements or no transitions). Consistently, they mentioned a significant correlation between higher FC in the pre‐SMA network and postoperative improvement. Table 3 demonstrates detailed information on the results of fMRI studies.

**TABLE 3:** A summary of findings of studies on DTI.

Study	Field strength	Covariates	Studied tracts/regions	FA (fractional anisotropy)	MD (mean diffusivity)	ad (axial diffusivity)	RD (radial diffusivity)
Avila‐Funes, 2016	1.5 T	Age, sex, educational level, myocardial infarction, angina pectoris, stroke or hypercholesterolaemia, hypertension, diabetes, smoking status, cognitive status	Whole brain	Frail individuals had lower FA in extensive areas of WM	Frail individuals had higher MD in extensive areas of WM	Frail individuals had higher ad in extensive areas of WM	Frail individuals had higher RD in extensive areas of WM
Ducca, 2022	3 T	Age, sex, race, educational levels, hypertension, coronary artery disease, diabetes, current smoking, stroke, cognitive status, body mass index, APOEe4 status	The superior longitudinal fasciculus and posterior limb of the internal capsule, as well as the genu, body and splenium of the corpus callosum	No significant difference among cognitively normal participants	Frailty status was associated with lower gMD	No significant difference among cognitively normal participants	No significant difference among cognitively normal participants
Maltais, 2020	—	Age, sex, educational level, diabetes mellitus, hypertension, hypercholesterolaemia, cognitive status, short physical performance battery (lower extremity functioning)	The corona radiata, internal capsule, corpus callosum, external capsule, thalamic radiation, occipital fasciculus, longitudinal fasciculus	No significant difference	Increased in anterior limb of internal capsule, external capsule, posterior corona radiata, posterior thalamic radiation, superior corona radiata, superior frontal occipital fasciculus and superior longitudinal fasciculus among frail participants	Increased AD in the anterior limb of internal capsule, external capsule, posterior corona radiata, posterior thalamic radiation, superior corona radiata, superior frontal occipital fasciculus and superior longitudinal fasciculus among frail participants	Increased in anterior limb of internal capsule, external capsule, posterior corona radiata, posterior thalamic radiation, superior corona radiata, superior frontal occipital fasciculus and superior longitudinal fasciculus among frail participants
Tian, 2020	—	Age, sex, race, educational level, APOEe4 status, BMI, cardiovascular disease	Whole brain	Higher frail score was associated with lower FA of the body of the corpus callosum and superior fronto‐occipital fasciculus ‐ The frail group had lower FA of the body of the corpus callosum and superior fronto‐occipital fasciculus at a marginal significance. ‐ Compared to the nonfrail group, the prefrail group had lower FA of the superior fronto‐occipital fasciculus and a trend toward lower FA of the body of the corpus callosum	‐ A higher frail score was associated with higher MD of the medial frontal cortex, putamen, caudate, thalamus and anterior cingulate cortex ‐ Compared to the nonfrail group, the frail group had higher MD of the medial frontal cortex, putamen, caudate, thalamus and anterior cingulate cortex ‐ Compared to the nonfrail group, the prefrail group had significantly higher MD of the putamen	No significant difference	No significant difference
Gutiérrez‐Zúñiga, 2023	3 T	Age, sex, medical history (number of regular medications, vascular diseases, cancer and smoking history), physical exercise in metabolic equivalents and quality of life measured by the CASP‐12 scale	—	With higher FI there was a decrease in FA in corpus callosum and superior longitudinal fasciculus and anterior corona radiata in both hemispheres	With higher FI there was an increase in MD in corpus callosum and superior longitudinal fasciculus and anterior corona radiata in both hemispheres	Not examined	Not examined

Abbreviations: AD, axial diffusivity; APOE e4, apolipoprotein e4; BMI, body mass index; DTI, diffusion tensor imaging; FA, fractional anisotropy; gFA, general factors for FA; gMD, general factors for MD; MD, mean diffusivity; RD, radial diffusivity; ROI, region of interest; WM, white matter; WMH, white matter hyperintensities.

#### 3.4.3 DTI Studies

Among the four DTI papers, two studies showed lower FA [25, 44], and two [25, 37] reported higher ad and higher RD. Three of these studies [25, 37, 44] showed increased MD in the frail group. Avila‐Funes et al. [25] reported a decreased FA in the corpus callosum (CC), anterior limb of the internal capsule (ALIC), external capsule (EC) and posterior thalamic radiation (PTR) in the frail group. Furthermore, two [25, 37] studies reported an increased MD in their tracts of interest, such as CC, ALIC, EC, PTR, SFOF (superior fronto‐occipital fasciculus), posterior and superior corona radiata and superior longitudinal fasciculus. Gutiérrez‐Zúñiga et al. [44] also reported lower FA and higher MD in the CC, superior longitudinal fasciculus and anterior corona radiata on both sides. Regarding ad and RD, both of the studies by Maltais et al. [37] and Avila‐Funes et al. [25] observed higher ad and RD values in the ALIC, EC and PTR. The other two DTI studies by Ducca et al. [28] and Tian et al. [41] excluded MCI participants. Ducca et al. [28] did not observe any significant differences regarding the DTI parameters of FA, ad and RD. However, they mentioned that frailty status was associated with lower global MD in their cognitively normal participants [28]. On the other hand, Tian et al. [41] noted lower FA in the SFOF and the body of the CC in both frail and prefrail groups and a significant association with higher frailty scores. Furthermore, they observed higher MD in CC, ALIC, EC, PTR, SFOF, posterior and superior corona radiata and superior longitudinal fasciculus [41]. More detailed information on DTI results can be obtained from Table 4.

**TABLE 4:** A summary of findings of studies on fMRI.

Study	Tesla sequence	Brain areas	Connectivity/activation	Covariates	Results
Lammers, 2020	3 T	SMA, pre‐SMA	Rs‐FC	Sex, age, max. functional independence, ASA score, Cad, CHF, malignoma, diabetes, smoking status, nutritional status, CCI, BMI, cognitive status, depressive symptoms, albumin, NT‐proBNP	There was a notable distinction in functional connectivity within the SMA between robust (nonfrail) patients and frail and prefrail patients, whereas no significant difference was observed between frail and prefrail patients. Frail patients had the lowest functional connectivity compared to functionally robust patients. There was a notable distinction in functional connectivity within the SMA between robust (nonfrail) patients and frail and prefrail patients, whereas no significant difference was observed between frail and prefrail patients. Frail patients had the lowest functional connectivity compared to functionally robust patients. There was no significant difference in functional connectivity between correlated and anticorrelated brain regions among frail patients. No correlations were observed between frailty and functional connectivity in the network prior to SMA. Functional connectivity in the pre‐SMA network was found to be significantly cortical region dependent and notably diminished in subjects with higher MMSE scores among the confounding variables. Functional connectivity was found to be associated with GPT performance alone, when correlated brain regions were examined individually; functional robustness and frailty were not influenced. Functional connectivity in anticorrelated regions of the SMA network was similarly associated with TMT‐A and partial performance but not with functional robustness or frailty
Lammers, 2022	3 T	SMA, pre‐SMA	Rs‐FC	Sex, age, BMI, cognitive status, nutritional status, CCI, ASA score, stroke, malignant disease, albumin, NT‐proBNP, creatinine, haemoglobin, anaesthesia type, type of surgery, length of hospital stay, ICU admission rate, length of ICU stay, presence of postoperative complications, severity of surgical complication, presence of postoperative delirium	The authors of the study examined 120 cognitively normal patients which were undergoing various elective surgeries. Fourteen days prior to and 3 months subsequent to the procedure, functional neuroimaging changes were evaluated by introducing dynamic frailty states. After surgery, physical improvement was noted in 19 patients, progression to (pre)frailty was documented in 24 patients and no noticeable shift was found in 77 individuals. Postoperative transition types that were more unfavourable were related with decreased functional connectivity in the pre‐SMA network. An exploratory analysis suggested that the association was restricted to patients who were prefrail at baseline. In the initial analysis, no correlation was found between the kind of transition and the functional connectivity of SMAs. Transition from prefrailty to robustness was linked with greater functional connectivity in an exploratory analysis, and advancement in robust patients was associated with greater SMA network segmentation. Dysfunctions of cortical networks implicated in higher cognitive control of mobility are related with postoperative transitions between frailty stages, according to the findings. As part of strategies to avoid frailty, neurofeedback or brain stimulation may be utilised to target the pre‐SMA
Suárez‐Méndez, 2020	0.23 T	Key areas for motor function and the central processing of proprioception (i.e., SMG, SPL, AG and sensorimotor cortex (precentral and postcentral gyri))	Rs‐FC	Age, gender, functional status, neuropsychological status	After correction for multiple comparisons, three significant clusters of FC differences emerged: two associated with seeds situated in the right hemisphere (AG and SPL) and one pertaining to the bilateral SMG. The direction of FC disruptions was consistent: the frail group consistently displayed diminished synchronization between the clusters and the seeds in the upper beta frequency band. To begin with, a diminished FC was observed in the vicinity of the right AG and various regions, such as the right motor cortex, precuneus, posterior cingulate cortex and sensorimotor cortex (precentral and postcentral gyri) Additionally, a diminished frontal cortex (FC) is observed in the vicinity of the right SPL and the anterior regions of the left hemisphere, including the frontal pole, the anterior cingulate, the lateral and medial frontal areas (e.g., premotor cortex) and the left temporal pole In conclusion, FC was diminished in the bilateral cingulate cortex, areas of the sensorimotor cortex, the right precuneus, and the left premotor cortex (IFG and FMG), SMA and the bilateral SMG. In the alpha, mu, and low beta frequency bands, as well as when the sensorimotor cortex was employed as a seed, no statistically significant variations were observed In comparison to their robust counterparts, the intranetwork mean FC was overall lower among frail older adults. To be more specific, participants who were frail demonstrated a diminished FC in the upper beta band across the FPN, pDMN and VAN. Moreover, within the upper beta band, the DAN exhibited a strong trend in the same direction (i.e., toward a lower FC in the frail group)

Abbreviations: ASA, American Society of Anaesthesiologists; BMI, body mass index; Cad, coronary artery disease; CCI, Charlson comorbidity index; CHF, congestive heart failure; FC, functional connectivity; fMRI, functional magnetic resonance imaging; GPT, Grooved Pegboard Test; N/A, not applicable; NT‐proBNP, N‐terminal pro‐brain natriuretic peptide; Rs, resting state; SMA, supplementary motor area; TMT‐A, Trail‐Making‐Test Part A.

#### 3.4.4 PET Studies

One of the included studies performed PET scan to evaluate frailty status, as measured by FI. Maltais et al. [36] illustrated that mean cortical beta‐amyloid level was not associated with FI, but the accumulation of the beta‐amyloid in the anterior and posterior putamen and precuneus region significantly correlated with frailty and its severity. More detailed information on PET results can be obtained from Table 5.

**TABLE 5:** A summary of findings of the study on PET scan.

Study	Analysis details	Covariates	Results
Maltais, 2019	PET data gathering begun 50 min after [18F]‐florbetapir was injected at a mean rate of 4 MBq/kg weight. The mean duration from the PET examination to the clinical assessment in close proximity to the PET was 76.3 days (standard deviation: 40.6)	Age, gender, educational level, cognitive status, MAPT group allocation, ApoE e4 genotype	The mean cortical Aβ load was not found to be associated with a FI derived from noncognitive measures. Nevertheless, in community‐dwelling older adults, Aβ in both the anterior and posterior putamen was found to be associated with FI severity in both cross‐sectional and prospective studies. Furthermore, the progressive accumulation of Aβ in the precuneus region was found to be correlated with a heightened severity of frailty. Frailty following an incident was not associated with cortical or regional Aβ. This research demonstrated that cerebral Aβ accumulation may influence the progression of frailty in elderly individuals over time

Abbreviations: ApoE e4, apolipoprotein E e4; FI, frailty index; MAPT, Multidomain Alzheimer's Preventive Trial; PET, positron emission tomography; SUVR, standard uptake value ratios.

#### 3.4.5 Differences in Neuroimaging Findings Between the Two Diagnostic Instruments for Frailty

Four studies [36, 38, 39, 44] used FI as an instrument for assessment of the frail individuals. Three of them [38, 39, 44] reported MRI results. Siejka et al. in both their studies [38, 39] discussed the changes in WMH and microbleeds, and their findings regarding the association between these findings and FI and frailty progression were almost similar to other studies. Gutiérrez‐Zúñiga et al. [44] observed changes in total cortex volume which was negatively correlated with FI. Although this observation was in line with other studies’ findings, the correlation of total cortex volume with other scores of frailty was not mentioned in other studies. On the other hand, Maltais et al. [36] reported PET results and no other study was available to compare its results with.

## 4 Discussion

As inferred from the findings of this investigation, notable distinctions between frail and robust groups were identified through MRI findings, primarily illustrating an increased count and volumes of WHMs and reduced GMV, compared to the robust group, in some brain regions including the cerebellum, hippocampi, middle frontal gyri, right inferior parietal lobule, precentral gyrus, left insula, anterior cingulate and middle occipital gyrus, indicative of vascular changes and brain atrophy, both of which contribute to cognitive impairment and even dementia [46]. Also, a significantly lower TIV was reported in one study [31]. In future investigations, adjusting for TIV will help to identify the individual GMV changes in frail older adults. This might partly explain the inconsistent results of the different studies. The sole prospective study addressing the correlation between beta‐amyloid load in specific brain regions and frailty revealed a positive association between heightened Aβ accumulation in the precuneus cortex, an Alzheimer's disease‐specific region, and posterior putamen, suggesting a potential trend toward Alzheimer disease in advanced stages of frailty [36]. Additionally, four studies [25, 28, 37, 41] employing DTI underscored the correlation between frailty and the integrity of both grey and WM microstructures, revealing inconsistent outcomes across various investigations, but mostly showing higher MD, lower or unchanged FA and higher or unchanged RD and ad, mostly in corpus callosum, thalamic radiations, corona radiate, internal and external capsule and some association tracts. These important findings in nondemented participants can aid investigators in better understanding the neuroimaging underpinnings of frailty that may lead to cognitive impairment, as well as the implications of frailty on brain health. Also, the shared neuroimaging findings in frailty can be a guide for early detection of preclinical neurological disease such as Alzheimer disease and vascular dementia in individuals with frailty.

Such pattern of changes is in line with changes in Alzheimer disease and might indicate predisposition of frail individuals to Alzheimer's disease [47, 48]. Moreover, the investigations conducted by Lammers et al. in 2020 and 2022, comprising two fMRI studies, revealed a discernible decrease in FC associated with the progression of the frailty syndrome in the SMA network but not in the pre‐SMA network [32, 33]. Additionally, Mendez et al. found reduced FC between the right angular gyrus and right motor, precuneus, posterior cingulate and sensorimotor cortices in the frail group [30]. Such FC changes in the default‐mode network especially the precuneus and posterior cingulate cortex are similar to Alzheimer's disease [49]. Prior MEG studies reported diminished power and FC in primary sensorimotor, pre‐ and supplementary motor areas, coupled with augmented connectivity in higher cognitive resting‐state networks in individuals exhibiting frailty [30, 40].

As discussed earlier in this article, PF is a unique systemic clinical syndrome with a biological basis that influences various aspects of the body best described by Fried criteria [1]. The correlation of this phenomenon with both the structure and functional changes in the brain, as elucidated in the studies included in this systematic review, is significant and would not have been anticipated without a profound understanding of the underlying biological processes. Recently, the correlation between PF and cognitive decline has gained more attention in medical research [50, 51, 52]. Recent studies have revealed the distinct association between this phenomenon and cognitive dysfunction, even without having overt dementia [51, 53]. In line with the primary objective of this article, the investigation of neuroimaging changes in nondemented older adults experiencing frailty enables us to clarify the brain structure alterations in PF and, consequently, on the cognitive status of these individuals.

By excluding papers that exclusively centred on patients with dementia or those unwilling to eliminate participants who might have dementia, we tried to minimise the impact of neurodegenerative disorders on the brain and obtain a focused perspective on discerning the specific relationship between frailty syndrome and the brain alterations, independent of neurodegenerative diseases or dementia. However, early stages of dementia, such as those observed in patients with MCI, may have minimal impacts on our findings. Nevertheless, as discussed in most of the included studies, the reported findings are common among the majority of participants, with frailty being the only consistent feature among them. In addition, by excluding individuals with dementia, the results of this systematic review may provide more insight on the chronological relationship between frailty and dementia, providing some level of confidence that frailty may be a precursor to dementia, rather than being the result of it.

Jiang et al. conducted a comprehensive study involving more than 483,000 participants, revealing a significant correlation between the degree of frailty and higher levels of total WMH, as well as reduced GMV [54]. In a separate study conducted by Zúñiga et al., fMRIs of 347 patients were analysed. The researchers identified 204 edges that had a positive correlation with the FI and 188 edges that exhibited a negative correlation [55]. Both of these articles did not exclude patients with dementia. Future study would be enhanced by using stricter patient selection criteria, particularly by eliminating patients with dementia, in order to assure the validity and interpretability of findings.

Various articles have proposed two distinct pathophysiological pathways to explain the observed brain damage in the frail older adults. The observations in the frail population predominantly propose a shared foundational origin for these phenomena—systemic vascular damage. While the precise role of vascular diseases in frailty syndrome remains less established, some evidence in the literature suggests a potential cause‐and‐effect relationship between the two. Avila‐Funes et al. identified that disturbances in the structure of carotid arteries were more prevalent in frail individuals compared to nonfrail counterparts [56]. Newman et al. discovered that, among those without a history of cardiovascular disease (CVD), the extent of underlying CVD, as measured by carotid ultrasound and ankle–arm index, along with left ventricular hypertrophy assessed through ECG and echocardiography, exhibited a correlation with frailty [57]. Besides, some new studies had shown a positive relationship between retinal microvascular damages and PF [58, 59]. Consistent with this, the predominant MRI finding in the included studies appears to be an elevation in both the number and size of WMHs within frail groups, findings in line with vascular pathology [60]. Cortical brain infarcts, as linked with PF as shown by Kant et al. [31], serve as an MRI marker of large vessel disease and exhibit an association with cognitive decline [61]. All these pieces of evidence collectively suggest a common basis between PF and neurological damage, seemingly rooted in vascular physiopathology. Prior research indicates a distinct association between different presentations of vascular disorders and dementia [62, 63]. These manifestations have a shared characteristic in the form of small blood vessel alterations, indicating a connection between frailty and dementia. To comprehensively evaluate these findings, further investigations with a larger number of participants are warranted.

Another suggested pathway explores the influence of inflammatory and oxidative stress factors in the frailty syndrome on the brain [64, 65]. Two recent studies establish a correlation between higher proinflammatory markers and an elevated risk of frailty and cognitive frailty [66, 67]. The presence of these factors as a link between this syndrome and neuroimaging changes necessitates further comprehensive investigations in this domain.

Such pathophysiological pathways contribute to cognitive impairment, as well as vascular and Alzheimer dementia. Given the shared neuroimaging findings with alzheimer's disease, such as brain atrophy especially in the hippocampus and precuneus cortex, higher WMHs and CMBs, WM microstructural changes in similar tracts as alzheimer's disease, reduced FC in DMN and importantly higher amyloid deposition in the precuneus cortex, it can be inferred that frailty is a preclinical manifestation of Alzheimer disease. Also, the vascular compromise, as shown by higher WMHs, is significant in frailty and can further contribute to cognitive impairment and even progression to dementia [68].

Our findings contribute to the understanding of frailty in nondemented individuals by emphasizing its multifaceted nature and the significance of various diagnostic criteria, such as gait speed and grip strength. These measures not only serve as indicators of physical health but may also correlate with neuroimaging findings related to cognitive function and overall brain health. Future research should further investigate the intricate relationships between neuroimaging results and frailty to enhance diagnostic and intervention strategies.

We had several limitations in our systematic review which should be considered in order to make a fair conclusion based on the results. Although most of the included studies recruited Fried criteria as their main frailty measurement, diversity in assessment tools might have affected the categorization of participants and their frailty stage due to the focus of the measurement tool on either cognitive of physical aspects of the condition. Besides, the small number of fMRI and PET studies makes it challenging to make a proper conclusion based on their results. Therefore, more multimodal neuroimaging studies and a uniform assessment tool for frailty should be conducted to clarify the unknown aspects of neurophysiological changes in nondemented frailty. Also, to determine whether or not frailty can be a risk factor or precursor for developing Alzheimer dementia, longitudinal studies especially with amyloid PET scans are warranted. Besides, we included studies with nondemented frail participants in this systematic review. However, except for two studies [28, 41], the majority of included studies did not exclude MCI participants [3, 25, 26, 27, 29, 30, 31, 32, 33, 34, 35, 36, 37, 38, 39, 40, 42, 43, 44, 45]. This could be due to the fact that frailty is a risk factor for MCI and is prevalent among MCI cases [52]. As MCI could potentially affect the neuroimaging findings in the study participants [3, 25, 26, 27, 29, 30, 31, 32, 33, 34, 35, 36, 37, 38, 39, 40, 42, 43, 44, 45], we strongly recommend the future studies to either exclude MCI participants or assess imaging findings separately in nondemented frail individuals with MCI and those who are cognitively normal, to be able to provide a more specific interpretation on the neuroimaging correlates of frailty in cognitively normal individuals.

To conclude, significant neuroimaging distinctions between frail and robust individuals were noted, emphasizing vascular changes in brain tissue, altered beta‐amyloid accumulation and grey and WM microstructure changes. The correlation between PF and cognitive decline is earlier discussed in earlier studies, even without overt dementia. Systematic vascular damage and inflammatory/oxidative stress are two common pathophysiological pathways suggesting a link between PF and neurological damage. The study underscores the importance of understanding both the brain structure and function of PF, offering insights into potential pathways for cognitive decline in nondemented older adults.

## Ethics Statement

The authors have nothing to report.

## Conflicts of Interest

The authors declare no conflicts of interest. Dr. Raji is supported in his research by NIA grants R01AG072637 and R01AG079241.

## Supporting information


**Table S1** Search term for each database.

**Table S2** Quality assessment of the included case–control studies.

**Table S3** Quality assessment of the included cohort studies.

**Table S4** Publication bias risk assessment of the included studies.
