# Supplementary material for: Neuroimaging Findings in Nondemented Frail Individuals: A Systematic Review
Source: J Cachexia Sarcopenia Muscle. 2025 Feb 11;16(1):e13719. doi: 10.1002/jcsm.13719 (PMC11813630; doi:10.1002/jcsm.13719)
Supplement: Supplementary file 1 — Table S1 Search term for each database. [file JCSM-16-e13719-s001.docx]

**Table S1.** Search term for each database

| 1. **PubMed:** **778** (30 Jan 2024) |
| --- |
| ("frail elderly"[Title/Abstract] OR "frailty"[Title/Abstract] OR "frail*"[Title] OR "Frailties" [Title/Abstract] OR "Frailness"[Title/Abstract] OR "Frailty Syndrome"[Title/Abstract] OR "Debility" [Title/Abstract] OR "Debilities"[Title/Abstract]) AND ("neuroimaging"[MeSH Terms] OR MRI OR MR OR fMRI OR magnetic resonance imaging OR "neuroimaging" OR "Diffusion Tensor Imaging" OR "Diffusion Weighted Imaging" OR "Brain Mapping" OR connectome OR "Cerebral Angiography" OR "Cerebral Ventriculography" OR Myelography OR Pneumoencephalography OR "Positron-Emission Tomography"[Mesh] OR PET Scan OR “tau pet imaging”) |
| **2. Embase: 826** (30 Jan 2024) |
| ('frail elderly'/exp OR 'frailty'/exp OR 'frailty syndrome'/exp OR 'oligophrenia'/exp OR 'frailty index'/exp OR 'frail scale'/exp OR 'frailty score'/exp OR 'frailty index score'/exp) AND ('neuroimaging'/exp OR 'nuclear magnetic resonance imaging'/exp OR 'functional magnetic resonance imaging'/exp OR 'diffusion tensor imaging'/exp OR 'diffusion weighted imaging'/exp OR 'brain mapping'/exp OR 'connectome'/exp OR 'brain angiography'/exp OR 'brain ventriculography'/exp OR 'positron emission tomography'/exp OR 'myelography'/exp OR 'pneumoencephalography'/exp) |
| **3. Other sources (searching manually): 3** |
