# Supplementary material for: Neuroimaging Findings in Nondemented Frail Individuals: A Systematic Review
Source: J Cachexia Sarcopenia Muscle. 2025 Feb 11;16(1):e13719. doi: 10.1002/jcsm.13719 (PMC11813630; doi:10.1002/jcsm.13719)
Supplement: Supplementary file 2 — Table S2 Quality assessment of the included case–control studies. [file JCSM-16-e13719-s003.docx]

**Table S2: Quality assessment of the included case-control studies**

|  | Selection | | | | | Comparability | | | Exposure | | | Score |
| --- | --- | --- | --- | --- | --- | --- | --- | --- | --- | --- | --- | --- |
| **Study** | Case definition adequacy | Representativeness of the cases | Selection of controls | Definition of controls | Subtotal | Age | Sex | Subtotal | Ascertainment of exposure | Non-response rate | Subtotal |  |
| Suárez-Méndez, 2020 | * | * |  | * | 3 | * | * | 2 | * |  | 1 | 6 |
| Chunmei Li, 2021 | * | * |  | * | 3 | * | * | 2 | * |  | 1 | 6 |
| Mathieu Maltais, 2019 | * | * |  | * | 3 |  |  | 0 | * |  | 1 | 4 |
| Mathieu Maltais, 2020 | * | * |  | * | 3 |  |  | 0 | * |  | 1 | 4 |
| Sugimoto, 2019 | * | * |  | * | 3 |  | * | 1 | * |  | 1 | 5 |
| Tian, 2020 | * |  | * | * | 3 | * | * | 2 | * |  | 1 | 6 |
