# Supplementary material for: Neuroimaging Findings in Nondemented Frail Individuals: A Systematic Review
Source: J Cachexia Sarcopenia Muscle. 2025 Feb 11;16(1):e13719. doi: 10.1002/jcsm.13719 (PMC11813630; doi:10.1002/jcsm.13719)
Supplement: Supplementary file 3 — Table S3 Quality assessment of the included cohort studies. [file JCSM-16-e13719-s004.docx]

**Table S3: Quality assessment of the included cohort studies**

| **Study** | Selection | | | | | Comparability | | | Outcome | | | | Score | |
| --- | --- | --- | --- | --- | --- | --- | --- | --- | --- | --- | --- | --- | --- | --- |
|  | Representativeness of the exposed cohort | Selection of the non-exposed cohort | Ascertainment of Exposure | Demonstration that outcome of interest was not present at start of study | Subtotal | Age | Sex | Subtotal | Assessment of outcome | Follow up length adequacy | Adequacy of follow up | Subtotal |  |  |
| Avila-Funes, 2016 |  | * | * |  | 2 | * | * | 2 | * | * |  | 2 | 6 |  |
| Chen, 2015 | * | * |  | * | 4 | * | * | 2 | * | * | * | 3 | 9 |  |
| Chunga, 2016 | * | * | * | * | 4 | * | * | 2 | * | * | * | 3 | 9 |  |
| Emma L. Ducca, 2022 | * | * | * |  | 3 | * | * | 2 | * | * |  | 2 | 7 |  |
| M. J. Kant, 2019 |  | * | * | * | 3 | * | * | 2 | * |  |  | 1 | 6 |  |
| M.J. Kant, 2018 | * | * | * | * | 4 | * | * | 2 | * |  |  | 1 | 7 |  |
| Lammers, 2020 |  | * | * |  | 2 | * | * | 2 | * |  |  | 1 | 5 |  |
| Lammers, 2022 |  | * | * | * | 3 | * | * | 2 | * |  |  | 1 | 6 |  |
| Mathieu Maltais, 2019 |  | * | * |  | 2 | * |  | 1 | * |  | * | 2 | 5 |  |
| Siejka, 2020 | * | * | * | * | 4 | * | * | 2 | * | * |  | 2 | 8 |  |
| Siejka, 2017 | * | * | * | * | 4 | * | * | 2 | * | * | * | 3 | 9 |  |
| SOURDET, 2021 |  | * | * | * | 3 | * | * | 2 | * | * |  | 2 | 7 |  |
| Suárez-Méndez, 2021 |  | * | * | * | 3 | * | * | 2 | * |  |  | 1 | 6 |  |
| Nishita, 2019 | * | * | * | * | 4 | * | * | 2 | * | * |  | 2 | 8 |  |
| Zhao, 2021 | * | * | * |  | 3 | * | * | 2 | * |  | * | 2 | 7 |  |
| Isernia, 2023 | * | * |  | * | 3 | * | * | 2 | * | * |  | 2 | 7 |  |
| Gutiérrez-Zúñiga, 2023 | * | * |  | * | 3 | * | * | 2 | * |  | * | 2 | 7 |  |
