# Supplementary material for: Neuroimaging Findings in Nondemented Frail Individuals: A Systematic Review
Source: J Cachexia Sarcopenia Muscle. 2025 Feb 11;16(1):e13719. doi: 10.1002/jcsm.13719 (PMC11813630; doi:10.1002/jcsm.13719)
Supplement: Supplementary file 4 — Table S4 Publication bias risk assessment of the included studies. [file JCSM-16-e13719-s002.docx]

| Study | Selection bias | | Performance bias | Attrition bias | Detection bias | | | | Reporting bias |
| --- | --- | --- | --- | --- | --- | --- | --- | --- | --- |
|  | Q1 | Q2 | Q3 | Q4 | Q5 | Q6 | Q7 | Q8 | Q9 |
| Avila-Funes, 2016 | Yes | Yes | Yes | No | No | Yes | Yes | Yes | No |
| Chen, 2015 | Yes | Yes | Yes | No | No | Yes | Yes | Yes | No |
| Chunga, 2016 | Yes | Yes | Yes | No | Yes | Yes | Yes | No | No |
| Emma L. Ducca, 2022 | Yes | Yes | Yes | No | No | Yes | Yes | No | No |
| M. J. Kant, 2019 | Yes | Yes | Yes | No | No | Yes | Yes | Yes | No |
| Suárez-Méndez, 2020 | Yes | Yes | Yes | No | No | Yes | Yes | No | No |
| M.J. Kant, 2018 | Yes | Yes | Yes | No | No | Yes | Yes | Yes | No |
| Lammers, 2020 | Yes | Yes | Yes | No | No | Yes | Yes | Yes | No |
| Lammers, 2022 | Yes | Yes | Yes | No | No | Yes | Yes | Yes | No |
| Chunmei Li, 2021 | Yes | Yes | Yes | No | No | Yes | Yes | Yes | No |
| Mathieu Maltais, 2019 | Yes | Yes | Yes | No | No | Yes | Yes | Yes | No |
| Mathieu Maltais, 2019 | Yes | Yes | Yes | No | No | Yes | Yes | Yes | No |
| Mathieu Maltais, 2020 | Yes | Yes | Yes | Yes | No | Yes | Yes | Yes | Yes |
| Siejka, 2020 | Yes | Yes | Yes | Yes | No | Yes | Yes | Yes | No |
| Siejka, 2017 | Yes | Yes | Yes | Yes | No | Yes | Yes | No | No |
| SOURDET, 2021 | Yes | No | Yes | No | No | Yes | Yes | No | Yes |
| Suárez-Méndez, 2021 | Yes | No | Yes | No | No | Yes | Yes | No | No |
| Sugimoto, 2019 | Yes | Yes | Yes | No | No | Yes | Yes | Yes | No |
| Tian, 2020 | Yes | Yes | Yes | No | No | Yes | Yes | Yes | Yes |
| Nishita, 2019 | Yes | Yes | Yes | No | No | Yes | Yes | Yes | No |
| Zhao, 2021 | Yes | Yes | Yes | No | Yes | Yes | Yes | Yes | No |
| Isernia, 2023 | Yes | Yes | Yes | Yes | No | Yes | Yes | Yes | Yes |
| Gutiérrez-Zúñiga, 2023 | Yes | Yes | No | Yes | No | Yes | Yes | Yes | Yes |

**Table S4: Publication bias risk assessment of the included studies.**
